# ﻿Two new species of the spider genus *Songthela* (Mesothelae, Liphistiidae) from Hunan Province, China

**DOI:** 10.3897/zookeys.1241.146992

**Published:** 2025-06-16

**Authors:** Shoushuo Han, Yan Zhang, Daiqin Li, Xin Xu

**Affiliations:** 1 College of Life Sciences, Hunan Normal University, Changsha 410081, Hunan Province, China Hunan Normal University Changsha China; 2 Centre for Behavioural Ecology and Evolution (CBEE) & Arachnid Resources Centre of Hubei, School of Life Sciences, Hubei University, 368 Youyi Road, Wuhan 430062, Hubei Province, China Hubei University Wuhan China

**Keywords:** Araneae, barcode gene, *bispina*-group, *COI*, description, morphology, taxonomy, trapdoor spiders, *unispina*-group

## Abstract

Two new species of the primitively segmented spider genus *Songthela* Ono, 2000, which were collected from Hunan Province, China, are described based on specimens of both sexes: *S.dongta***sp. nov.** (♂♀), and *S.lixi***sp. nov.** (♂♀). *Songtheladongta***sp. nov.** is assigned to the *bispina*-group, while *S.lixi***sp. nov.** belongs to the *unispina*-group, based on the morphology of male palps and female genitalia. We also provide mitochondrial cytochrome *c* oxidase subunit I (COI) sequences for species identification and calculate the intra- and interspecific genetic distance among these two new species and 26 known *Songthela* species. These molecular data highlight distinct genetic divergence between the two new species and their congeners, facilitating future species delimitation. This study not only expands the known diversity of *Songthela*, but also contributes to a growing framework for understanding biogeographic patterns and evolutionary processes in ancient spider lineages.

## ﻿Introduction

**Figure 1.**
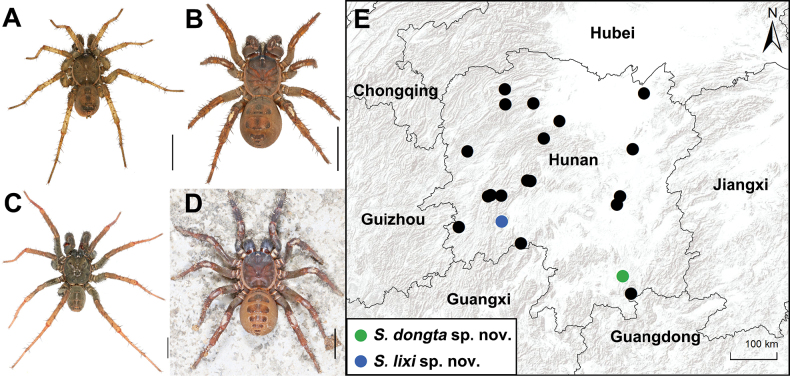
General somatic morphology and collection localities of two new *Songthela* species **A, C** male **B, D** female **A, B***Songtheladongta* sp. nov. **C, D***Songthelalixi* sp. nov. **A** XUX-2023-017 **B** XUX-2023-016 **C** XUX-2022-240 **D** XUX-2022-244 **E** Map showing the type localities of the two new and 23 known *Songthela* species (black dots) in Hunan Province, China. Scale bar: 5 mm.

The primitively segmented spider genus *Songthela* Ono, 2000 belongs to the suborder Mesothelae within the family Liphistiidae Thorell, 1869. Members of this genus retain plesiomorphic arachnid characters, such as abdominal tergites (Fig. 1A–D) and spinnerets located in the ventral median portion of the abdomen (Bristowe 1975; Haupt 2003; Schwendinger and Ono 2011; Xu et al. 2015). Currently, *Songthela* contains 38 valid species, primarily distributed across southern China (Chongqing, Guizhou, Hubei, Hunan, Sichuan, Yunnan, Zhejiang), with one species, *S.sapana* (Ono, 2010), reported from northern Vietnam (World Spider Catalog 2025). Of these, 23 species are found in Hunan Province, China (Fig. 1E).

**Figure 2.**
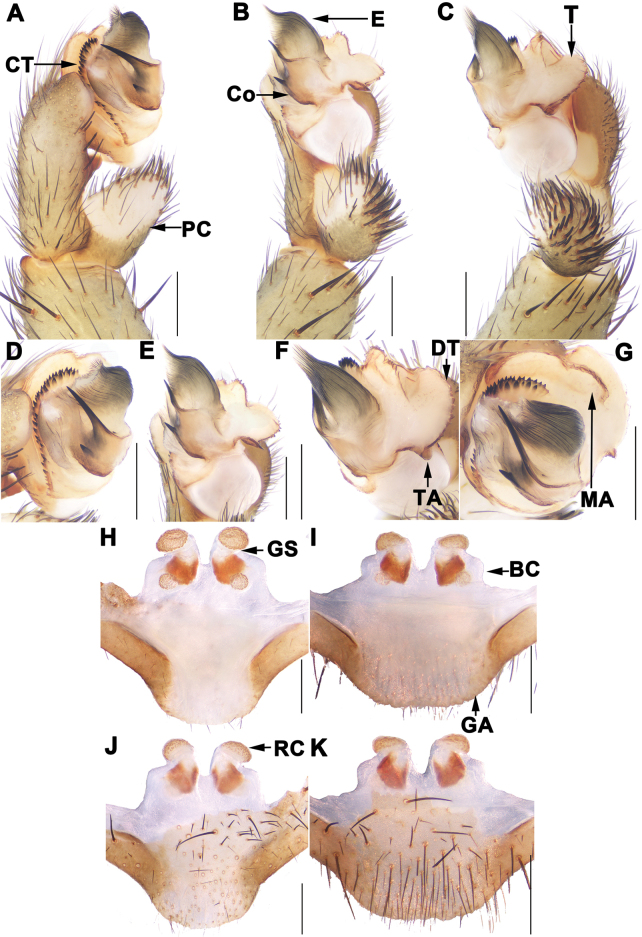
Male palp and female genitalia of *Songtheladongta* sp. nov. **A, D** palp, prolateral view **B, E** palp, ventral view **C, F** palp, retrolateral view **G** palp, distal view **H, I** vulva, dorsal view **J, K** vulva, ventral view **A–G** XUX-2023-017 **H, J** XUX-2023-014 **I, K** XUX-2023-016. Scale bars: 0.5 mm.

**Figure 3.**
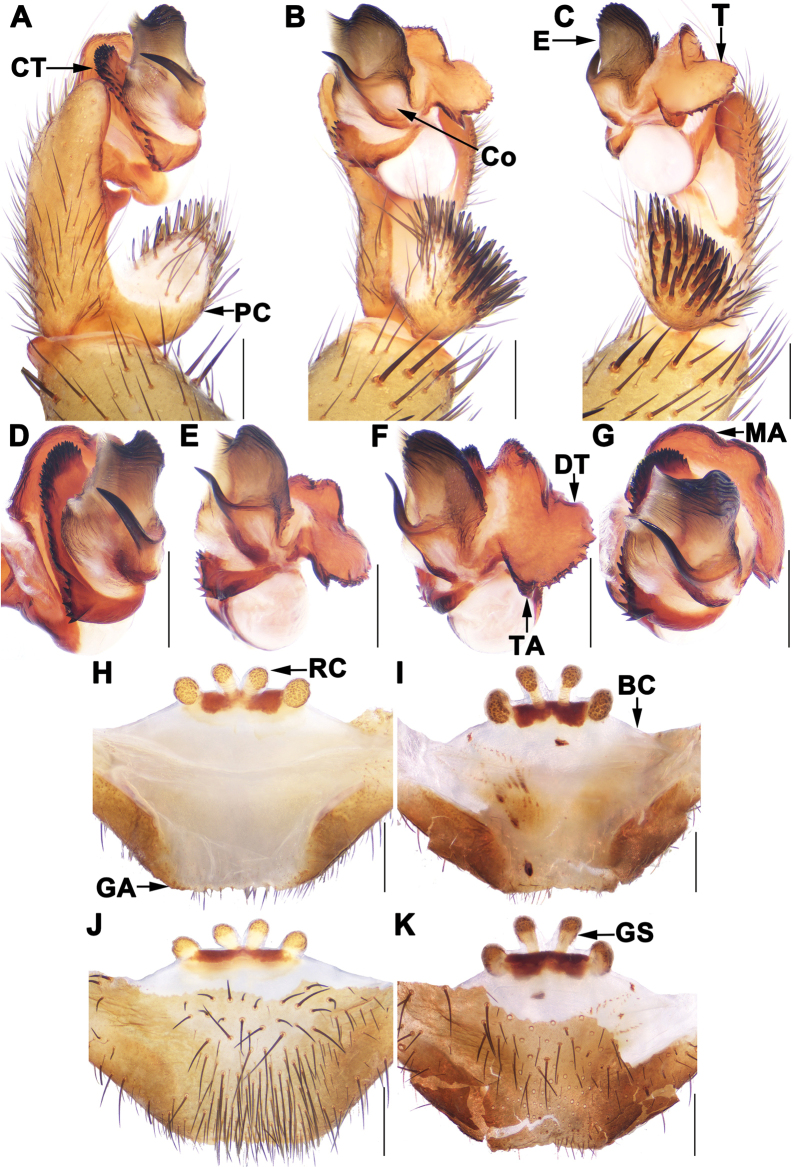
Male palp and female genitalia of *Songthelalixi* sp. nov. **A, D** palp, prolateral view **B, E** palp, ventral view **C, F** palp, retrolateral view **G** palp, distal view **H, I** vulvae, dorsal view **J, K** vulvae, ventral view **A–C** XUX-2022-241 **D–G** XUX-2022-240 **H, J** XUX-2022-239 **I, K** XUX-2022-244. Scale bars: 0.5 mm.

Most *Songthela* species can be assigned to three species groups: the *bispina*-group, the *multidentata*-group, and the *unispina*-group, based on morphological characters and molecular data (Li et al. 2022). Species in the *bispina*-group feature a smooth palpal conductor with two apical spines, and their lateral receptacular clusters are situated on the dorsal wall of the bursa copulatrix (Fig. 2). The *multidentata*-group is characterized by a palpal conductor with one apical spine and several teeth on its middle portion, lateral receptacular clusters situated on the dorsal wall, and a sclerotised posterior margin of the bursa copulatrix (figs 11, 12 in Li et al. 2022). In contrast, species in the *unispina*-group have a smooth palpal conductor with one apical spine, lateral receptacular clusters positioned nearly the anterior margin of the bursa copulatrix, and lack a sclerotised posterior margin (Fig. 3).

In this study, we diagnose and describe two new species of the genus *Songthela* from Hunan Province, China, based on the morphology of the male palps and female genitalia. Moreover, we provide cytochrome *c* oxidase subunit I (*COI*) sequences for the two new species to facilitate future identification, and calculate the genetic distances among the two new species and 26 known *Songthela* species.

## ﻿Material and methods

We collected the specimens alive from Hunan Province, China (Fig. 1E), and transported subadults to the laboratory, where they were reared until maturity. For adult specimens, the right four legs were removed, preserved in absolute ethanol, and stored at –80 °C for DNA extraction. The remaining parts of each specimen were preserved in 80% ethanol and retained as vouchers for morphological examination. All voucher specimens are deposited at the School of Life Sciences, Hubei University, Wuhan, Hubei Province, China.

We examined and dissected the specimens using an Olympus SZ61 stereomicroscope. The soft tissues of female genitalia were removed and digested using 10 mg/ml pancreatin (Biosharp Company, Hefei, Anhui, China) for at least three hours at room temperature. Male palps and female genitalia were observed and photographed using a digital CCD camera (Kuy nice E3ISPM, China) mounted on an Olympus BX53 compound microscope. Compound-focused images were generated using Helicon Focus v. 6.7.1. All measurements are given in millimeters. Leg and palp measurements are presented in the following order: leg total length (femur, patella, tibia, metatarsus, tarsus) and palp total length (femur, patella, tibia, tarsus).

We extracted total genome DNA from spider leg muscles using the Animal Genomic DNA Isolation Kit (Kangwei Biotech, China) following the manufacturer’s protocol, amplified *COI* using the primer pair LCO1490/HCO2198 (Folmer et al. 1994) following standard protocols (Li et al. 2020). We retrieved *COI* sequences of 26 known *Songthela* species (one per species) from the National Center for Biotechnology Information (NCBI) and aligned the *COI* data matrix using Geneious Prime 2022 (https://www.geneious.com) with gap opening/extension penalties set to 24/3. We calculated the genetic distances using MEGA 11 (Tamura et al. 2021) based on Kimura 2-parameter (K2P) and *p*-distance nucleotide substitution models.

Abbreviations used are: ALE = anterior lateral eyes; AME = anterior median eyes; BC = bursa copulatrix; BL = body length; CL = carapace length; Co = conductor; CT = contrategulum; CW = carapace width; DT = dorsal extension of terminal apophysis of tegulum; E = embolus; GA = genital area; GS = genital stalk; MA = marginal apophysis of tegulum; OL = opisthosoma length; OW = opisthosoma width; PC = paracymbium; PLE = posterior lateral eyes; PME = posterior median eyes; RC = receptacular cluster; T = tegulum; TA = terminal apophysis of tegulum.

## ﻿Taxonomy

**Family Liphistiidae Thorell, 1869**

**Genus *Songthela* Ono, 2000**

### ﻿ Songthela dongta

Taxon classificationAnimaliaAraneaeLiphistiidae

Han & Xu sp. nov.

7CC7D7FA-09DF-5C82-B855-855AFEC3C164

https://zoobank.org/DAFFB852-2817-4072-A7E0-1F9B50D96CB5

Figs 1 , 2

#### Type material.

***Holotype***: China • ♂; Hunan Province, Chenzhou City, Guiyang County, Dongta Park; 25.74°N, 112.74°E; alt. 379 m; 29 July 2023; X. Xu, Y.X. Li, Y.C. Xiong, S.S. Han leg.; XUX-2023-017. ***Paratypes***: China • 2 ♀♀; same data as for the holotype; XUX-2023-014, 016.

#### Diagnosis.

Male palp of *S.dongta* sp. nov. resembles those of *S.goulouensis* Yin, 2001 and *S.zizhu* Li, Chen, Liu, Li & Xu, 2022, by conductor with long upper apical spine, but can be distinguished from *S.goulouensis* by conductor with wider base (Fig. 2B, E vs. figs 45, 46 in Xu et al. 2015), and slightly larger terminal apophysis of tegulum (Fig. 2F vs. fig. 46 in Xu et al. 2015); from *S.zizhu* by contrategulum with two rows of serrated edge (Fig. 2A, D vs. fig. 8a, d in Li et al. 2022), and larger marginal apophysis of tegulum (Fig. 2G vs. fig. 8g in Li et al. 2022). Female genitalia of *S.dongta* sp. nov. resemble that of *S.aokoulong* Li, Chen, Liu, Li & Xu, 2022 by middle genital stalks slightly tilted outward, but can be distinguished from the latter by shorter middle genital stalks and the lateral receptacular clusters far away from the base of middle receptacular clusters (Fig. 2H–K vs. fig. 4h–o in Li et al. 2022).

#### Description.

Male (holotype; Fig. 1A). Carapace yellowish brown; opisthosoma blackish brown, with 12 dark brown tergites attached a pair of thick bristles, the 2^nd^ to 6^th^ larger than others and the 5^th^ largest; sternum narrow, longer than wide; ocular area slightly raised with several pointed hairs; chelicerae robust with promargin of cheliceral groove with 10 denticles of variable size; each leg with three claws and strong setae and spines; 7 spinnerets. Measurements: BL 9.37, CL 4.44, CW 3.87, OL 4.33, OW 3.30; ALE > PLE > PME > AME; leg I 13.15 (3.77, 1.73, 2.73, 3.13, 1.79), leg II 13.65 (3.63, 1.56, 2.78, 3.49, 2.19), leg III 14.59 (3.75, 1.84, 2.61, 4.20, 2.19), leg IV 19.92 (4.78, 2.09, 3.81, 6.12, 3.12).

***Palp*.** Paracymbium unpigmented and unsclerotised in prolateral view, with several setae and spines on the tip (Fig. 2A). Contrategulum with two rows of small and densely serrated edge (Fig. 2D, G). Tegulum with a slightly helicoid marginal apophysis, a slightly helicoid dorsal extension of terminal apophysis, and a thumb-like terminal apophysis retrolaterally (Fig. 2C, F). Conductor smooth with two spines, the longer spine pointed to the middle of opening of embolus, the shorter spine located in the middle portion, the base fused with embolus (Fig. 2B–E). Embolus largely sclerotised with a wide opening, with several longitudinal ribs reaching tip in retrolateral view (Fig. 2E–G).

**Female** (XUX-2023-014). Carapace yellowish brown; opisthosoma light brown, with 12 blackish-brown tergites attached a pair of thick bristles, the 2^nd^ to 5^th^ larger than others and the 4^th^ largest; sternum and ocular area similar as male; chelicerae robust with promargin of cheliceral groove with 12 denticles of variable size; each leg with three claws and strong setae and spines; 7 spinnerets. Measurements: BL 13.98, CL 6.55, CW 5.55, OL 6.29, OW 5.19; ALE > PLE > PME > AME; palp 11.25 (3.92, 2.25, 2.26, 2.82), leg I 12.56 (4.04, 2.31, 2.34, 2.50, 1.37), leg II 12.33 (3.69, 2.21, 2.29, 2.63, 1.51), leg III 13.67 (3.87, 2.29, 2.45, 3.35, 1.71), leg IV 19.93 (5.61, 2.98, 3.43, 5.34, 2.57).

***Female genitalia*.** The middle receptacular clusters large, situated on the anterior margin of the bursa copulatrix; the lateral ones small, situated on the dorsal wall of the bursa copulatrix; the middle ones with obvious stalks, tilted outward; the posterior margin of the genital area slightly arc-shaped (Fig. 2H–K).

#### Variation.

Females vary in body size. Measurements for females (*N* = 2) are as follows: BL 13.39–13.98, CL 5.65–6.55, CW 4.82–5.55, OL 6.29–6.63, OW5.19–5.44.

#### Etymology.

The species epithet, a noun in apposition, refers to the type locality.

#### Distribution.

Hunan (Chenzhou), China

#### GenBank accession number.

XUX-2023-014: PV330146; XUX-2023-016: PV330147; XUX-2023-017: PV330148.

#### Remarks.

The maximum and mean intraspecific genetic distances of *S.dongta* sp. nov. are 0.15% and 0.1%, respectively, based on both K2P and *p*-distance. The mean interspecific genetic distance between two new species is 13.71% (K2P) and 12.49% (*p*-distance). Among the 28 species analyzed, the closest interspecific genetic distance is 11.42% (K2P) and 10.47% (*p*-distance) between *S.dongta* sp. nov. and *S.huayanxi* (Table 1).

**Table 1.** Interspecific genetic distances among two new and 26 known *Songthela* species based on *COI* sequences. GenBank accession codes of 26 known species are provided in parentheses. The lower left and upper right matrices show the genetic distances (%) calculated using K2P and *p*-distance substitution model, respectively.

	Species	1	2	3	4	5	6	7	8	9	10	11	12	13	14	15	16	17	18	19	20	21	22	23	24	25	26	27	28
1	*S.dongta* sp. nov.		12.59	10.47	10.62	17.30	11.23	11.08	15.93	10.47	15.93	13.66	16.84	12.29	15.48	16.54	15.02	18.51	11.53	16.69	18.36	12.75	15.78	18.21	11.76	16.84	18.06	15.93	10.91
2	*S.lixi* sp. nov.	13.85		13.66	14.87	15.02	15.63	14.57	17.15	13.51	15.78	6.83	15.33	14.57	15.17	15.33	8.65	15.48	15.78	13.81	16.54	10.77	15.48	15.93	14.89	13.96	15.93	14.87	15.10
3	*S.aokoulong* (OK351173)	11.44	15.23		10.17	17.00	12.90	9.86	16.84	9.56	16.24	13.96	15.78	8.95	14.42	17.00	15.02	17.60	11.08	16.54	17.75	13.05	15.17	17.15	9.38	15.63	16.84	14.57	12.92
4	*S.bispina* (OK351255)	11.59	16.74	11.12		17.30	12.29	10.93	17.00	10.47	16.24	13.35	17.15	11.53	15.48	17.15	14.26	18.21	10.47	18.06	18.36	13.51	15.93	17.15	12.32	14.87	17.60	17.15	12.25
5	*S.dapo* (OK351079)	19.90	16.86	19.53	19.87		18.21	17.00	18.06	15.63	15.48	15.63	9.10	16.39	17.75	14.42	16.54	13.51	18.06	16.24	10.93	16.39	16.84	10.47	17.46	9.41	8.95	16.39	16.28
6	*S.goulouensis* (MT102211)	12.39	17.66	14.37	13.67	21.17		13.81	19.58	13.20	17.30	15.48	17.60	12.90	17.00	17.15	15.78	19.73	14.42	18.97	19.88	15.48	17.15	19.73	15.26	17.75	18.51	16.39	12.08
7	*S.hangzhouensis* (KP229843)	12.14	16.39	10.68	12.00	19.57	15.54		17.30	4.10	16.84	13.96	15.78	7.44	15.48	17.45	14.26	18.21	12.59	15.78	16.84	14.11	15.78	16.69	5.88	16.39	15.17	15.78	12.92
8	*S.huangyang* (MT102213)	18.04	19.65	19.20	19.42	20.89	23.00	19.79		16.84	15.78	16.08	18.06	17.30	15.78	16.39	16.84	16.69	16.54	13.20	17.60	14.57	16.24	17.45	18.75	17.30	18.21	16.69	18.12
9	*S.huayanxi* (OK351128)	11.42	15.02	10.33	11.44	17.75	14.79	4.25	19.18		15.63	13.51	15.63	6.37	14.72	16.39	14.87	18.06	11.23	15.78	16.08	13.20	15.78	16.39	4.60	15.48	15.78	15.48	12.75
10	*S.jinyun* (OL982296)	18.09	17.82	18.52	18.47	17.50	19.92	19.31	17.80	17.70		15.93	15.17	16.08	13.20	16.39	16.84	16.24	18.06	15.93	16.54	16.24	14.87	15.93	16.54	15.17	16.08	15.17	16.78
11	*S.lianhe* (OK351151)	15.22	7.24	15.62	14.86	17.65	17.52	15.65	18.22	15.06	18.04		16.08	15.02	14.87	14.57	7.44	15.63	15.48	14.57	16.39	11.08	14.42	15.17	15.07	13.81	15.63	14.72	15.44
12	*S.lingshang* (OK351121)	19.21	17.24	17.85	19.65	9.87	20.25	17.88	20.91	17.65	17.03	18.22		16.08	17.15	13.51	16.08	14.11	17.30	15.93	8.04	15.48	16.69	11.84	16.91	10.02	5.61	15.17	16.78
13	*S.liui* (MW450989)	13.68	16.36	9.65	12.76	18.81	14.40	7.94	19.84	6.74	18.31	16.98	18.31		16.08	18.06	15.63	17.60	11.38	16.84	16.24	13.35	15.63	17.00	6.80	15.78	15.33	14.87	13.59
14	*S.longbao* (OL982299)	17.46	17.07	16.10	17.52	20.44	19.47	17.48	17.82	16.47	14.64	16.65	19.62	18.26		16.24	15.78	16.54	16.84	15.63	17.15	15.17	13.66	17.15	17.65	14.26	17.00	12.14	15.77
15	*S.multidentata* (OK351081)	18.85	17.22	19.46	19.65	16.24	19.71	20.11	18.65	18.66	18.56	16.23	15.05	21.00	18.38		16.08	12.29	15.93	16.54	13.51	15.33	17.75	15.02	17.83	13.96	13.51	16.24	15.94
16	*S.pyriformis* (MN400625)	16.89	9.31	16.90	15.89	18.79	17.84	15.96	19.24	16.76	19.21	7.88	18.22	17.73	17.82	18.20		16.24	15.78	15.33	15.93	10.62	15.33	17.30	16.54	15.02	15.63	15.93	16.28
17	*S.serriformis* (OL982297)	21.57	17.53	20.34	21.17	15.23	23.32	21.19	19.05	20.98	18.46	17.70	15.91	20.38	18.87	13.53	18.52		17.91	15.48	12.59	16.39	18.82	13.81	18.93	11.99	13.20	16.69	16.44
18	*S.shuyuan* (MN400635)	12.79	18.00	12.23	11.49	21.02	16.51	14.09	18.82	12.39	21.04	17.62	19.90	12.58	19.25	18.05	17.91	20.73		18.06	17.15	14.57	15.17	17.45	10.85	16.24	17.15	15.48	11.41
19	*S.tianmen* (OK351193)	19.00	15.30	18.83	20.78	18.42	22.11	17.78	14.59	17.77	17.99	16.27	18.08	19.19	17.56	18.77	17.23	17.37	20.85		15.78	14.57	16.69	15.78	17.46	15.17	15.63	16.84	17.28
20	*S.tianzhu* (MW450988)	21.30	18.83	20.47	21.27	12.06	23.44	19.25	20.22	18.21	18.85	18.65	8.64	18.46	19.57	15.03	17.98	13.93	19.64	17.81		16.54	17.00	12.75	16.91	10.47	6.22	16.08	17.45
21	*S.unispina* (OK351164)	14.03	11.78	14.39	14.95	18.66	17.49	15.75	16.28	14.59	18.40	12.13	17.42	14.78	17.07	17.24	11.55	18.72	16.31	16.29	18.83		16.24	16.39	15.26	13.81	15.33	14.26	15.44
22	*S.wangerbao* (OL982298)	17.93	17.48	17.17	18.14	19.33	19.66	17.98	18.34	17.98	16.78	16.14	19.07	17.78	15.17	20.47	17.22	21.98	17.17	18.97	19.49	18.49		18.06	16.36	15.93	17.00	8.04	16.61
23	*S.xiangnan* (MT102212)	21.21	18.03	19.71	19.76	11.45	23.37	19.12	20.05	18.68	18.11	17.06	13.11	19.53	19.64	16.98	19.75	15.53	20.20	17.80	14.27	18.64	21.02		18.57	10.17	10.32	17.45	16.61
24	*S.xianningensis* (KP229827)	12.96	16.77	10.12	13.67	20.15	17.37	6.18	21.79	4.76	18.87	17.04	19.33	7.19	20.29	20.56	18.90	22.11	11.85	19.97	19.32	17.15	18.75	21.61		17.46	16.36	16.54	13.42
25	*S.xiujian* (OK351217)	19.28	15.53	17.69	16.73	10.14	20.47	18.68	19.84	17.46	17.04	15.34	10.85	17.92	15.90	15.60	16.81	13.18	18.47	17.00	11.38	15.38	18.04	11.02	20.07		9.71	15.48	16.11
26	*S.yuping* (MW450990)	20.88	18.07	19.28	20.27	9.71	21.51	17.08	21.12	17.85	18.24	17.66	5.90	17.30	19.42	15.05	17.60	14.76	19.69	17.65	6.59	17.27	19.52	11.26	18.58	10.51		16.24	16.44
27	*S.zimugang* (OK351256)	18.15	16.67	16.31	19.74	18.69	18.68	17.93	18.99	17.53	17.14	16.52	17.05	16.73	13.29	18.42	18.07	19.13	17.56	19.16	18.24	15.91	8.55	20.15	18.89	17.45	18.47		15.77
28	*S.zizhu* (OK351106)	11.97	17.03	14.53	13.65	18.62	13.45	14.47	21.00	14.29	19.22	17.51	19.25	15.41	17.83	18.15	18.55	18.79	12.73	19.78	20.12	17.48	19.10	19.02	15.06	18.31	18.79	17.98	

### ﻿ Songthela lixi

Taxon classificationAnimaliaAraneaeLiphistiidae

Zhang & Xu sp. nov.

E88929CF-91B9-5EBC-BAD6-10A0E01AEF0A

https://zoobank.org/8874460B-5488-4F9F-861F-4B8E8B2F9A09

Figs 1 , 3

#### Type material.

***Holotype***: China • ♂; Hunan Province, Shaoyang City, Suinng County, Lixi Town, Jiangtang Village; 26.79°N, 110.41°E; alt. 407 m; 24 August 2022; X. Xu, Y. Zhan, Y. Zhang, Y.X. Li leg.; XUX-2022-241. ***Paratypes***: China • 2 ♂♂ 3 ♀♀; same data as for the holotype; XUX-2022-239, 240, 242–244.

#### Diagnosis.

Male palp of *S.lixi* sp. nov. resembles those of *S.lianhe and S.unispina* by conductor with a thick apical spine, but can be distinguished from *S.lianhe* by contrategulum with larger teeth proximally (Fig. 3A, D vs. fig. 14d in Li et al. 2022), and by curved marginal apophysis of tegulum (Fig. 3G vs. fig. 14g in Li et al. 2022); from that of *S.unispina* by slightly smaller terminal apophysis of tegulum (Fig. 3C, F vs. fig. 17f in Li et al. 2022). Female genitalia of *S.lixi* sp. nov. resemble those of *S.lianhe* and *S.unispina* by four receptacular clusters situated nearly along the anterior margin of the bursa copulatrix, but differ from *S.lianhe* by middle receptacular clusters distinct smaller than the lateral ones, from *S.unispina* by longer middle genital stalks (Fig. 3H–K vs. figs 15, 17h–k in Li et al. 2022).

#### Description.

**Male** (holotype). Carapace yellowish brown; opisthosoma light brown, with 12 dark brown tergites, the 2^nd^ to 6^th^ larger than others and the 4^th^ largest; sternum narrow, longer than wide; ocular area slightly raised with several pointed hairs; chelicerae robust with promargin of cheliceral groove with 8 denticles of variable size; each leg with 3 claws, with strong setae and spines; 7 spinnerets. Measurements: BL 9.41, CL 4.40, CW 4.09, OL 3.96, OW 2.99; ALE > PLE > PME > AME; leg I 14.53 (4.07, 1.81, 2.90, 3.89, 1.86), leg II 14.79 (3.96, 1.79, 2.93, 4.09, 2.02), leg III 15.70 (3.76, 1.82, 2.96, 4.95, 2.21), leg IV 20.78 (5.24, 1.98, 4.07, 6.57, 2.92).

***Palp*.** Paracymbium unpigmented and unsclerotised in prolateral view, with several setae and spines on the tip (Fig. 3A–C). Contrategulum with a row of serrated edge (Fig. 3A, D). Tegulum with a helicoid curved marginal apophysis and dorsal extension of terminal apophysis, and with a thumb-like terminal apophysis (Fig. 3C, F, G). Conductor smooth, fused with embolus basally, with a slightly curved and long apical spine pointed to the one-third of opening of embolus proximally (Fig. 3A, B, D, G). Embolus highly sclerotised, with a wide smooth opening, with several longitudinal ribs reaching tip in retrolateral view (Fig. 3G).

**Female** (XUX-2022-239). Carapace yellowish brown; opisthosoma light brown, with 12 black brown tergites attached to a pair of thick bristles, the 2^nd^ to 6^th^ larger than others and the 4^th^ largest; sternum and ocular area similar as male; chelicerae robust with promargin of cheliceral groove with 12 denticles of variable size; each leg with three claws and strong setae hairs and spines; 7 spinnerets. Measurements: BL 11.64, CL 5.70, CW 5.00, OL 5.08, OW 4.15; ALE > PLE > PME > AME; palp 10.88 (3.80, 2.07, 2.35, 2.66), leg I 11.94 (3.85, 2.24, 2.16, 2.41, 1.28), leg II 11.33 (3.35, 2.06, 2.11, 2.31, 1.50), leg III 12.26 (3.57, 2.06, 2.08, 2.80, 1.75), leg IV 16.77 (4.61, 2.46, 3.00, 4.46, 2.24).

***Female genitalia*.** Four receptacular clusters situated on the anterior margin of the bursa copulatrix; the middle ones with distinct stalks and V-shaped; the lateral ones larger than the middle ones; the anterior margin of the bursa copulatrix arc-shaped, the posterior margin of the genital area trapezoidal (Fig. 3H–K).

#### Variation.

Males and females differ in body size and the number of cheliceral teeth. Measurements for males (*N* = 3) are as follows: BL 8.97–9.41, CL 4.40–4.62, CW 3.98–4.09, OL 3.83–4.12, OW 2.77–3.04. For females (*N* = 3), measurements are: BL 10.90–14.12, CL 5.18–6.02, CW 4.47–5.26, OL 4.78–7.20, OW 3.93–5.46. The number of cheliceral teeth ranges from 8 to 12 (*N* = 6). In addition, the middle receptacular clusters exhibit variation: they either have longer stalks and are widely separated from each other at the base (Fig. 3I) or possess shorter stalks that are closely positioned at the base (Fig. 3H).

#### Etymology.

The species epithet, a noun in apposition, refers to the type locality.

#### Distribution.

Hunan (Shaoyang), China

#### GenBank accession number.

XUX-2022-239: PV330149; XUX-2022-240: PV330150; XUX-2022-241: PV330151; XUX-2022-242: PV330152; XUX-2022-243: PV330153; XUX-2022-244: PV330154.

#### Remarks.

Both the maximum and mean intraspecific genetic distances of the new species are 0% based on K2P and *p*-distance. Among the 28 species analyzed, the closest interspecific genetic distance is 7.24% (P2P) and 6.83% (*p*-distance) between *S.lixi* sp. nov and *S.lianhe* (Table 1).

## ﻿Discussion

The discovery of *S.dongta* sp. nov. and *S.lixi* sp. nov. underscores the urgency of documenting biodiversity in East Asian hotspots, where cryptic lineages often remain undetected. Morphologically, these two new species align with the *bispina*-group and *unispina*-group, respectively, and their genetic divergence from each other (13.59%–13.78% K2P; 12.39%–12.54% *p*-distance) confirms their evolutionary distinctness. This integrative approach—pairing morphology with *COI* barcoding—exemplifies modern taxonomy’s capacity to revolve cryptic diversity in understudied taxa.

Hunan Province now hosts 62% of all known *Songthela* species, partitioned among three species groups, the *bispina*-group, the *multidentata*-group, and the *unispina*-group. The wide distribution of *bispina*- and *unispina*-group species contrasts with the restricted range of the *multidentata*-group (central/southwest Hunan; Li et al. 2022). *Songtheladongta* sp. nov. and *S.lixi* sp. nov. not only expand the genus’s diversity but also bridge gaps in its biogeographic coverage, suggesting fine-scale habitat partitioning shaped by Hunan’s complex topography.

These findings position Hunan as a critical refuge for *Songthela*, an ancient lineage vulnerable to habitat fragmentation. The region’s concentration of endemic species emphasizes its role as a living laboratory for studying mesothele evolution and biogeography.

## Supplementary Material

XML Treatment for Songthela dongta

XML Treatment for Songthela lixi

## ﻿Additional information

### Author ORCIDs

Xin Xu https://orcid.org/0000-0001-5632-6622

### Data availability

All of the data that support the findings of this study are available in the main text.
